# Virophages and Their Interactions with Giant Viruses and Host Cells

**DOI:** 10.3390/proteomes6020023

**Published:** 2018-05-22

**Authors:** Haitham Sobhy

**Affiliations:** Helmy Institute for Medical Sciences, Zewail City of Science and Technology, Ahmed Zewail Road, October Gardens, 6th of October City, Giza 12566, Egypt; haithamsobhy@gmail.com or hsobhy@zewailcity.edu.eg

**Keywords:** giant viruses, virophage, nucleocytoplasmic large DNA viruses (NCLDVs), protein-protein interactions, post translational modifications

## Abstract

Virophages are small dsDNA viruses that were first isolated in association with some giant viruses (GVs), and then found in metagenomics samples. They encode about 20–34 proteins. Some virophages share protein similarity with Maverick/Polinton transposons or are considered as a provirophage, whereas about half of the protein’s repertoire remain of unknown function. In this review, we aim to highlight the current understanding of the biology of virophages, as well as their interactions with giant viruses and host cells. Additionally, the virophage proteomes were analyzed to find the functional domains that distinguish each virophage. This bioinformatics analysis will benefit further experimental investigations to understand the protein-protein interactions between virophages, giant viruses, and host cells.

## 1. Introduction

The discovery of virophages was associated with the discovery of giant viruses (GVs) [1,2,3,4]. Giant viruses are characterized by their giant particle size (larger than 600 nm) and genome size (up to 2.5 Mb). Most of these large viruses are classified in the *Mimiviridae* family. The first virus discovered in this family was *Acanthamoeba polyphaga* mimivirus (APMV), followed by the discovery of other viruses, including *Acanthamoeba castellani* Mamavirus, (ACMV). Both APMV and ACMV are considered closely related with about a 1.2 Mb genome, and encode about 1000 proteins. The first virophage was isolated in 2008, the so-called Sputnik virophage, in association with Mamavirus that infect *Acanthamoeba* cells [1]. Since that time dozen of virophages have been characterized (Table 1). 

## 2. Virophage Biology 

Virophages are small double-stranded (ds) DNA viruses. Their 17–30 kb genome encodes 16 to 34 putative proteins. About half of the encoded proteins are ORFans and have unknown functions. Some of the encoded proteins are conserved within most of the virophages and could have crucial roles in viral replication, such as FtsK-HerA family DNA-packaging ATPase, retrovirus-like, integrase cysteine protease, primase-superfamily 3 helicase (S3H), a zinc-ribbon domain protein, and major (MCP) and minor (mCP) capsid proteins [2,3,4]. 

Sputnik was cultured in association with ACMV within amoeba cells [1]. The co-infection of the virophage with ACMV leads to a decrease in the number of the lysed host cells, compared to those observed during infection by the giant virus alone. This decrease could be due to reduction of ACMV progeny. Using electron microscopy, the abnormal morphology of the ACMV particles and capsid was observed. The abnormal morphology included the accumulation of several layer of capsid at one side (see [1]). Moreover, small particles (thought to be virophages) were observed inside ACMV [1]. The association between virophages and mimiviruses lead to the hypothesis of the existence of “giant virus infection”, i.e., a small virus (virophage) infects a larger one. To my knowledge, the observation of virophage particles within the giant viruses was not reported by further studies. Generally, a virus enters into a host cell to replicate and, therefore, virophages cannot replicate within giant viruses. It is obvious that both virophages and giant viruses depend on host cells to replicate. Therefore, the term “giant virus infection” may fail to explain the relationships between virophages and giant viruses and, thus, the term should be elucidated by further studies. 

After Sputnik, number of virophages were isolated (Table 1). Among the virophages, the Zamilon virophage (meaning “a colleague”, in Arabic) was isolated with the Mont1 virus from Tunisian soil [12]. It replicates in the presence of some members of *Mimiviridae* (e.g., the Mont1 virus and Moumouvirus), but fails to replicate in the presence of Mimivirus. Several other virophages have been isolated as well, including Mavirus, which is isolated in association with *Cafeteria roenbergensis* virus (CroV) [7], and shares genomic features with Maverick/Polinton (MP) transposons, such as encoding retrovirus-like integrase, helicase, and protein-primed DNA polymerase [7]. Additionally, a dozen virophages were isolated from metagenomic samples [5,6,8,10,11]. The exact hosts or associated viruses of the metagenomic virophages remain to be validated, but it is suggested that they are associated with giant viruses and infect marine phytoplankton, algae, or protists. To summarize, the association of virophages and giant viruses were reported in some virophages, but remain to be elucidated in other cases, such as (i) metagenomics samples, as well as (ii) the detection of antibodies to the Sputnik virophage (but not giant Mamavirus) in human blood samples [13]. 

At least three virophages were identified by electron microscopy as an icosahedron virus particle (see [2,14]). The structure of the Sputnik virophage was resolved showing that non-enveloped icosahedral viruses with approximately 70 nm in diameter and mushroom-like protrusions are attached to capsomers [14]. Mavirus and Zamilon could resemble the icosahedral structure of Sputnik. Of note, a virophage-like genome, but not a viral particle, was isolated with *Phaeocystis globosa* virus (PgV) that infect algae; it is a so-called PgV-associated virophage (PgVV) [9]. Since PgVV is devoted to structural proteins, except MCP, it is proposed that PgVV replicates as a linear plasmid-like genome “provirophage” that can be integrated into the PgV genome. Currently, virophages are classified by the International Committee on Taxonomy of Viruses (ICTV) as one family, so-called *Lavidaviridae* (“Lavida-” stands for large virus-dependent or -associated virus) that comprises Sputnikvirus and Mavirus genera [2]. 

The nature of virophages and the interactions with giant viruses were of interest to a number of previous studies. For example, the discovery of the virophages opened a question regarding whether or not a genome encapsulated by proteins can be considered as a virus particle [2]. Since some virophages are unable to propagate independently, it is suggested that virophages cannot be considered as *bona fide* viruses. Therefore, some researchers defined virophages as satellite viruses, provirophages, or gene transfer elements [2,15]. On the other hand, the virophages could be able to use the transcriptional machinery encoded by the giant viruses, and use the giant virus factory to replicate, which may resemble the nuclear replication of small dsDNA viruses. To summarize, although the biology of virophages is of great interest, the nature and life cycle of virophages remain to be extensively investigated. This article is an attempt to highlight the features of the virophage proteome that could have potential roles in cellular interactions of virophages. 

## 3. Interactions between Virophages and Host Cells/Giant Viruses 

The interactions between virophages, giant viruses, and the host cells deserve to be investigated by future studies. It is suggested that virophages invade the viral factories of giant viruses and the virophages use these factories as transcription sites to replicate. On the other hand, the method by which the virophages enter into the host cells is largely unknown. It is suggested that Mavirus enters independently (i.e., does not require CroV co-infection) via an endocytic pathway, whereas Sputnik might enter host cells in association with the giant virus via phagocytic-like mechanism. The precise mechanisms of virophage entry remains to be investigated by further research.

The interactions between virophages and GVs have been investigated by few studies. Among the first attempts was culturing Mimivirus (APMV) 150 times on germ-free amoeba, leading to the emergence of a new strain, the so-called M4 strain [16]. More than 150 Mimivirus genes are either deleted or split in the fiberless-M4 viruses. The decreased replication of the Sputnik virophage after co-culturing with the M4 strain suggested that Mimiviral fibers (which are highly antigenic) could play a role in virophage infection. i.e., Sputnik uses the fiber to enter host cells [16]. Furthermore, knocking-down Mimivirus fiber-associated proteins using short interfering RNAs led to the emergence of short fibered-Mimiviruses [17,18]. Co-culturing Sputnik with the knocked-down Mimiviruses lead to an increased replication of Sputnik [18]. The implications of these two experiments suggest that the deleted proteins, but not the fiber, could play a major role in virophage-giant virus interaction, as it will be discussed in the following section. 

A recent finding showed that the replication of Zamilon increased after silencing three mimivirus genes; R349 (ubiquitin-protein transferase and harbors a HECT domain), R350 (ATP-binding and helicase activity), and R354 (DNA binding and nuclease activity) [19] (see also [20,21]). In normal conditions Mimivirus resists Zamilon, i.e., Zamilon does not replicate in the presence of Mimivirus. This leads to proposing a CRISPR–Cas-like adaptive immunity that protects some strains of mimiviruses from Zamilon infection. Another interesting finding showed the ability of Mavirus to integrate its genome loci into host cells [22]. The CroV infection reactivates Mavirus and the host cell is then lysed and liberate both CroV and Mavirus. 

Open questions remain to be answered, such as how the host cell retains the antiviral or CRISPR memory [21], particularly after the lytic infection of giant viruses. Interestingly, in many organisms, the CRISPR system could have a function beyond a defense mechanism, and an organism cannot inflate its genome by integrating additional genome sequences forever [23]. An additional explanation is that the interactions between mimiviruses, virophages, and host cells are orchestrated through the proteins encoded by the viruses or host cell. However, over 60% of virophages’ proteins are ORFans and have unknown functions, which hurdle the identification of these signaling pathways. 

## 4. The Potential Roles of the Proteins in the Cellular Interactions of the Virophages

Proteins usually harbor short peptides (3–5 residues) or long protein domains (up to 30 residues) to perform certain function and, therefore, they are named as functional motifs, reviewed in [24]. Classically, the functional units and domains in the proteins were used to predict their functions and interactions. The proteins containing the same motifs are most likely to have the same function [24,25]. Therefore, we first determined the protein functional units that characterize each virophage, which helps in the prediction of the cellular interactions of the virophage’s proteins (see [25]). Then, a comparative proteomics analysis was performed to identify the potential proteins that might mediate the interactions between virophages (from one side), and giant viruses and host cells (from the other side). For example, it is expected that Zamilon encodes proteins that mediate its replication with the Mont1 giant virus (but not with Mimivirus). Similarity, the provirophage PgVV proteins could encode unique motifs to facilitate its replication. It is noteworthy that the original host of most of these small viruses remain unknown. For these reasons it is of great impact to identify the functional units in virophage proteomes to reveal the cellular interactions of virophages with the host cells. 

**Figure 1:**
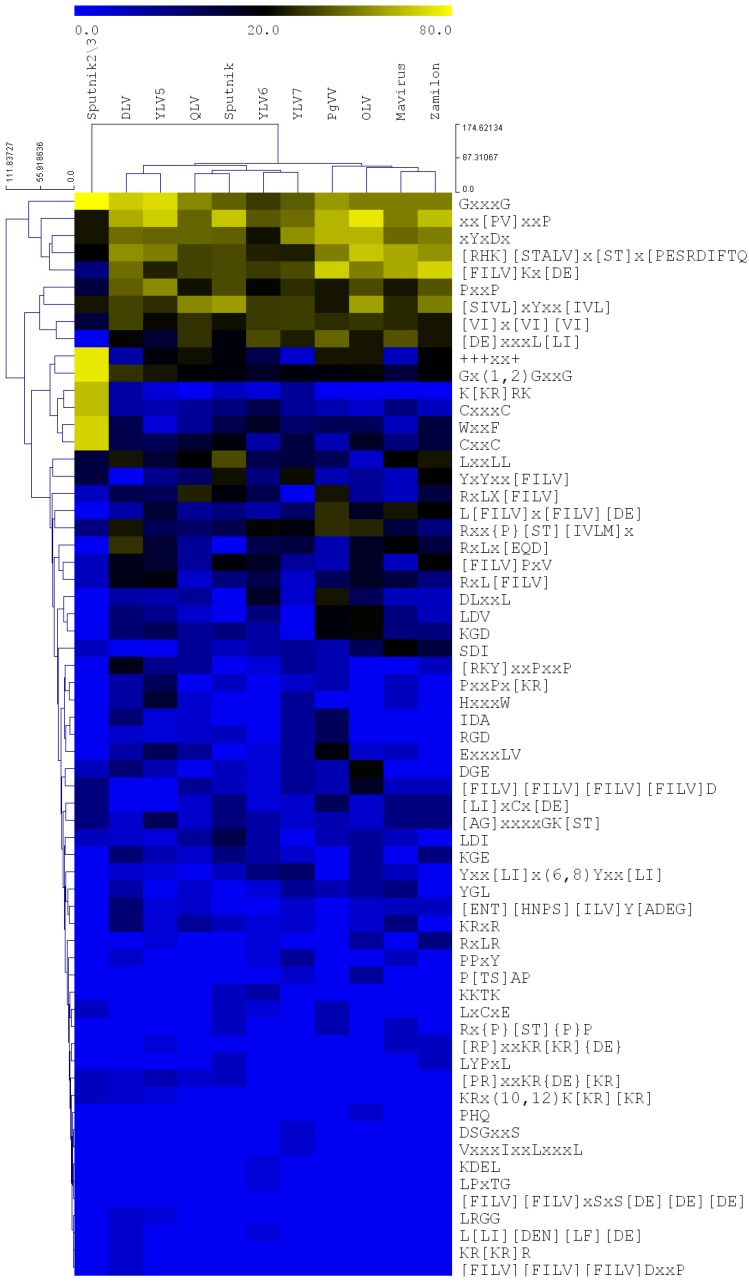
A hierarchical clustering (Euclidean distance and average linkage) heat map of the functional motif profile encoded by twelve virophages. The horizontal clusters represent the virophage species and the vertical clusters are the motifs. The nodes’ lengths are shown. The color scale is shown above the heat map, the blue color is 0%, i.e., absent in this proteome; whereas, the yellow color is 80%, i.e., all the proteins harbor at least one instance of this motif. The plot constructed from Table S1. For the list of proteins and motifs, see Supplementary Information SI-3.

In this analysis, a proteome-wide exact search for the functional motifs [25] was performed in the virophage proteomes (see supplementary method in the Supplementary file SI-1). The Spearman correlation was calculated to determine the proteomes that harbor similar functional motifs. The statistical analysis shows that the functional motifs profile [25] are different among the virophages, Figure 1, Table 2 and Table S1. For example, the correlation between the functional motif profile of YLV5 and DSLV have highest correlation (0.85). This suggests that YLV5 and DSLV could trigger similar cellular pathways within the host. In the same manner, OLV and YLV6, and Zamilon and QLV could trigger similar cellular interactions, whereas, Sputnik 2 and 3 are the divergent virophages (correlation < 0.6) that may trigger different cellular pathways. Previously, it was shown that the evolutionary-related poxviruses, or those that infect related hosts harbor similar functional motifs, as discussed in [24,25]. Therefore, it is suggested that the virophages with high correlation rank (i.e., that encode similar motifs) could acquire these sequences either from a common ancestor or from the same closely-related hosts. To understand the impact of these functional motifs on the interaction between virophages, giant viruses, and host replications and cells, we identified the motifs that distinguish each virophage and highlight their functions. 

Searching virophage proteomes for the functional motifs shows that about 70% of Zamilon proteins harbor the canonical sequence of the small ubiquitin-like modifier (SUMO)-binding motif (φKx[DE], where φ denotes large hydrophobic residues (F, I, L or V)), whereas about 38% of Sputnik proteins harbor the same motif (Table 3 and Table S1). The post-translational modification (PTM) processes (e.g., ubiquitination or SUMOylation) were reported as a major regulator of the replication of several other viral families. As previously reported, Zamilon replication was elevated (i.e., the highest replication fold number) after silencing the ubiquitin-related R349 protein (a HECT domain-containing ubiquitin transferase), even higher than R350 or R354 proteins [19]. Since the nucleases are core components of CRISPR-like immunity [19], it was expected that Zamilon replication could have been elevated after silencing R350 and R354 proteins, which is not the case here. Of note, human E3 ligases (harbor HECT domain) were shown to bind to human papillomavirus proteins, reviewed in [26]. This shows that PTM processes could play a major role in Zamilon replication. In support of this observation, Mimivirus R349 protein has been split in M4 isolate into R349a and R349b [16]. As mentioned above, the replication of Sputnik was decreased after co-culturing with M4. The possible explanation of the previous studies is that Sputnik depends on the PTM machinery of Mimivirus to hijack the cells and replicate, but Mimivirus R349 antagonizes Zamilon infection, i.e., superinfection exclusion of Zamilon. On the other hand, Mimivirus fibers have an antigenic effect on virophages. Once they are knocked down [17,18], virophages can replicate and propagate. To summarize, Mimivirus could depend on fiber and PTM-related proteins to exclude other viruses in host cells. 

Additionally, Sputnik and Mavirus proteins harbor immunoreceptor tyrosine-based activation motifs (ITAM), however Zamilon, PgVV, and QLV do not encode the same motif (Figure S1, Table 3 and Table S1, and Supplementary Information SI-2). ITAM gives a positive signal to the immune response. It is encoded by tumor viruses, including herpesviruses. It plays roles in viral latency [27,28,29], viral escape from immune response [30], suppression of apoptosis [31], or mediating the transformation of some cells [32]. The phosphorylation of the two tyrosine residues in ITAM facilitates its binding to Src homology 2 (SH2) domains-containing proteins [32], which then directs the proteins to ubiquitin-mediated proteasomal degradation [33]. ITAM is linked with acute pathogenesis, for example, it is encoded by the pathogenic strains, but not nonpathogenic, of hantaviruses [33]. In reoviruses, the phosphorylated ITAM recruits spleen tyrosine kinase (Syk) to virus factories [34].

Furthermore, PgVV does not harbor any known nuclear localization signal (NLS), whereas Zamilon encodes one protein that harbors a class 4 NLS motif. Noting that KR-rich motifs can be predicted using sequence search, which could function as NLS domain [35] (Table S2). Generally, the import and export of a protein into or from the nucleus is orchestrated by two motifs; NLS and nuclear export signal (NES), respectively, which have roles in viral nuclear trafficking and replication, reviewed in [24]. One possibility is that the nuclear shuttling is different between virophages, or PgVV could entirely replicate in the cytoplasmic virus factory. 

Taken together, virophages may depend on post-translational modification (PTM) processes (e.g., phosphorylation, ubiquitination, or SUMOylation) within the host cells. Moreover, the ITAM motif encoded by Sputnik and Mavirus, but not Zamilon, could explain why Zamilon fails to replicate in association with some giant viruses. 

## 5. Conclusions and Future Prospective

In this article, we reviewed the cellular interactions of virophages. Moreover, a comparative proteomic approach was used to predict the potential interactions between virophages, giant viruses, and host cells. The analysis highlights the role of PTM processes in virophage replication. It is of great interest to experimentally investigate the function of PTM- and ITAM-containing proteins in virophage-GV-cell interactions. The current bioinformatics analysis offers a dataset of candidate proteins (that could perform certain functions) for further experimental analyses. 

This bioinformatics analysis is consistent with the previous findings reported in other virus families, i.e., the roles of ITAM and PTM in the exclusion of other viruses or hijacking of the host cells. Noting that this analysis includes an exact data-mining search for experimentally validated motifs, which increases the possibilities of true positive results, unless the motif-containing proteins could evolve a new function [24,25]. As examples, short sequence motifs of 3–4 amino acids, such as RGD, PPxY, and PHQ, are encoded by a few virophages (Table S1). Similarly, virophages do not encode motifs that were previously described as a signature of other virus families, such as the adenovirus adhesion protein motif and polyomavirus agnoprotein motif. 

In conclusion, the relationship between virophages and giant viruses may not necessarily be a viral infection. Virophages could mutualistically remain latent in the host cell, such as Mavirus [22]. Virophages could also take advantage of the giant virus factory to replicate; and in this case the two viruses compete for the cellular resources, which leads to a decrease of the replication of the giant virus. The available data show that virophages could resemble to *bona fide* small DNA viruses (e.g., resemble to latency of herpesviruses or binding to E3 ligases as papillomaviruses). It is possible that some virophages enter into the host cells independent of the giant virus and they remain latent inside the cells. Once giant viruses infect the cells, the virophage replication is initiated. Virophages may share the resources of giant viruses, but may not benefit from infecting the giant virus itself, because both viruses depend on the host cell transcription machinery. On the other hand, the provirophage strains could be evolved to independent or semi-independent virus particles and, therefore, virophages may have a great impact on evolution of viruses. 

## Figures and Tables

**Table 1:** Virophages and the associated giant viruses, host cells, and genome sizes.

Virophage	Source	Giant Virus	Host	Genome Size (kb)	Number of ORFs	Reference
ALM *	Ace Lake, Antarctica			18	22	[5]
DSLV1 *	Dishui Lake, China			29	28	[6]
Mavirus	Coastal waters, USA	*Cafeteria roenbergensis* virus	Marine phagotrophic flagellate	19	20	[7]
OLV *	Organic Lake, Antarctica	Organic Lake phycodnavirus		26	24	[8]
PgVV	Dutch coastal waters	*Phaeocystis globosa* virus PgV-16T	Algae	20	16	[9]
QLV *	Qinghai Lake, China			23	25	[10]
Sputnik	Cooling tower water, France	Mamavirus	*A. polyphaga*	18	21	[1]
YSLV1 *	Yellowstone Lake, USA			28	26	[5]
YSLV2 *	Yellowstone Lake, USA			23	21	[5]
YSLV3 *	Yellowstone Lake, USA			27	23	[5]
YSLV4 *	Yellowstone Lake, USA			28	34	[5]
YSLV5 *	Yellowstone Lake, USA			30	32	[11]
YSLV6 *	Yellowstone Lake, USA			25	29	[11]
YSLV7 *	Yellowstone Lake, USA			23	26	[11]
Zamilon	Soil, Tunisia	Mont1 virus	*A. polyphaga*	17	20	[12]

* denotes isolated from metagenomics samples. ALM: Ace Lake Mavirus, DSLV: Dishui lake virophage, OLV: Organic Lake virophage, PgVV: Phaeocystis globosa virus virophage, QLV: Qinghai Lake virophage, YSLV: Yellowstone Lake virophage.

**Table 2:** Spearman rank correlation between the functional motifs among different virophages.

	DSLV	Mavirus	OLV	PgVV	QLV	Sputnik23	Sputnik	YLV5	YLV6	YLV7	Zamilon
DSLV	1										
Mavirus	0.681	1									
OLV	0.690	0.781	1								
PgVV	0.699	0.766	0.769	1							
QLV	0.764	0.805	0.791	0.833	1						
Sputnik23	0.470	0.513	0.563	0.538	0.638	1					
Sputnik	0.599	0.687	0.700	0.705	0.777	0.783	1				
YLV5	0.853	0.717	0.715	0.736	0.794	0.536	0.693	1			
YLV6	0.713	0.771	0.838	0.763	0.811	0.624	0.774	0.722	1		
YLV7	0.692	0.704	0.659	0.640	0.729	0.556	0.620	0.664	0.668	1	
Zamilon	0.661	0.782	0.805	0.741	0.838	0.629	0.785	0.757	0.819	0.657	1

**Table 3:** The motifs, their functions, and the percent of proteins that harbor the motif.

	DSLV	Mavirus	OLV	PgVV	QLV	Sputnik23	Sputnik	YLV5	YLV6	YLV7	Zamilon
**Function “Motif”**	**% of the Proteins**										
ISGylation, antiviral response “LRGG”	4	0	0	0	0	0	0	3	0	0	0
Protein ubiquitylation, and interaction with Nedd4 E3 ubiquitin ligases “PPxY”	4	5	0	0	0	0	0	0	3	8	0
SUMO binding to substrate “[FILV]Kx[DE]”	46	60	50	69	36	10	38	28	34	38	70
NLS motif “KRxR”	11	10	4	0	8	0	5	3	3	4	0
NLS motif—Bipartite “KRx(10,12)K[KR][KR]”	4	0	0	0	0	5	0	3	0	0	0
NLS motif—Class 1 “K[KR]RK”	7	0	0	0	0	65	5	3	3	8	0
NLS motif—Class 1 “KR[KR]R”	4	0	0	0	0	0	0	0	0	0	0
NLS motif—Class 2 “[PR]xxKR{DE}[KR]”	4	0	0	0	4	5	5	6	0	0	0
NLS motif—Class 4 “[RP]xxKR[KR]{DE}”	0	5	0	0	0	0	0	3	0	0	5
ITAM motif, positive signal of immune receptors “Yxx[LI]x(6,8)Yxx[LI]”	4	5	8	0	0	0	5	3	10	12	0

## Supplementary Materials

The following are available online at http://www.mdpi.com/2227-7382/6/2/23/s1, Supplementary information (SI-1): It contains the supplementary materials and methods, figures, and tables. Table S1: The functional motif profile table of motif-containing proteins encoded by virophages, Table S2: The predicted KR-rich motifs (a predicted NLS motif) in PgVV, Table S3: The coverage of each residue in the whole proteome.
