# Supplementary material for: Virophages and Their Interactions with Giant Viruses and Host Cells
Source: Proteomes. 2018 May 22;6(2):23. doi: 10.3390/proteomes6020023 (PMC6027436; doi:10.3390/proteomes6020023)
Supplement: Supplementary file 1 [file proteomes-06-00023-s001.zip › SI files/SI-1.pdf]

Virophages and their interactions with giant viruses and host cells

Haitham Sobhy

Supplementary Information – SI-1 (this document): It contains supplementary material and methods, supplementary tables and supplementary figures.

Supplementary Information – SI-2 (Excel file): It contains lists of the motif-containing proteins in each virophage proteome.

Supplementary Materials and Methods

The protein sequences of twelve virophages were downloaded from NCBI GenBank database. Only virophages of complete genomes (as in June, 2017) were downloaded. The virophage names and GenBank IDs are listed in table S1. The twelve virophages are Mavirus, Sputnik, Sputnik 2 and 3, Zamilon, Phaeocystis globosa virus virophage (PgVV), Dishui lake virophage 1 (DSLV), Organic Lake virophage (OLV), Qinghai Lake virophage (QLV) and Yellowstone Lake virophage 5, 6 and 7 (YSLV5, -6 and -7, respectively). The fasta file formatted protein sequences were processed by Shetti-Motif tool (Sobhy 2017), as suggested in the user manual. The tool performs exact text-mining search for built-in 130+ functional motifs that previously experimentally validated for other viruses, supplementary information SI-1. The list of the motifs and their functions are previously reviewed in (Sobhy 2016, Sobhy 2017), table S1. The output file, which contains the number of motif-containing proteins (MCPs) in each virophage, were then collected and a collective table was constructed, table S1. To calculate enrichment of the MCPs in a proteome, the numbers of MCPs were calculated then normalized (i.e. percent) to the total number of the proteins encoded by a virophage. If a protein harbors multiple sets of the same motif, only one instance is considered.

For statistical analysis, Spearman rank correlations was calculated using R statistics tool (https://www.r-project.org/), table 1. The heat-map and clustering based on the Euclidean distances and average linkage was performed using MeV tool (http://mev.tm4.org/), Fig. 1.

References

Nguyen Ba, A. N., et al. (2009). "NLStradamus: a simple Hidden Markov Model for nuclear localization signal prediction." BMC Bioinformatics **10**: 202.

Sobhy, H. (2016). "A Review of Functional Motifs Utilized by Viruses." Proteomes **4**(1): 3.

Sobhy, H. (2017). "A bioinformatics pipeline to search functional motifs within whole-proteome data: a case study of poxviruses." Virus Genes **53**(2): 173-178.

Supplementary Figures

Figure S1. Examples of ITAM motif sequences (Yxx[IL]x_{6-12}Yxx[IL], InterPro ID: IPR012316) in Sputnik and Mavirus virophages.

The protein sequences (in FASTA format) are listed below. The ITAM motif is in a bold font, underlined and highlighted in yellow. In Sputnik, the ITAM is flanked by the $Yx_{2-3}L$ motif (in bold and underlined), which is known as a signature of ITAM motif and used during virus budding and egress. The V9 protein is a Leucine- and tyrosine-rich protein (about 14% and 11% of the proteins length, respectively). For the full list of the motif sequence, see SI-2. For additional information regarding the amino acid coverage within virophages' proteins, see table S3 and Figure S2

>ACF16993.1|V9 Sputnik

1 MKELKYYEKVALSNFDILEMLDNKAEIVLYPNLIKYETID 40
41 DVLGPYGACVLLFEAKKNYGHWCCLFKREDNSIEFFNSYG 80
81 GYPDNSLK**YIPL**H**YREISNQYYPYLSL**LLLK**YPHKL**YYNE 120
121 FKFQKRANDIRTCGRWCVLRLLLKHLDIYEFKKYVDDMCS 160
161 YYKVTPDELVTMITI 175

>ADZ16416.1|hypothetical protein Mavirus

1 MKQYIWLNETIKSNKQLAGPRGSYKRPVSVDIFRSSTILD 40
41 PDKNYLLIVEEFHLHKIRLPLFKPAGHDYQVGIFNRSTDE 80
81 IMGVREVDFSTFVDEDGYMYDYVDVGTAINETLAGLCDGI 120
121 IGEEDIPVFSFNKHSKKFEITTTENFRNGHFIMFNDDMRV 160
161 DFNSFEFDDIDEEYSLVILNEDVETQDASTLEFLTPISHI 200
201 VIESNDLPVSYELLPSISKNTTISDNTGVFLTN**YKYLQQN** 240
241 **NQDYNSI**LFRVENSSNKYHNILQTNFNRFNLSFTIYDYDN 280
281 EKHPLTLLPQTVIQLKLLFESID 303

Figure S2. The average of the amino acid composition (abundance or coverage) within virophage proteins.

First, the coverage of the amino acid residues (within each protein) to the total length of the protein were calculated. The proteome averages were then calculated. The plot shows the residues versus the average coverage within the proteome. The error bars refer to standard deviation. The plot were constructed from Table S2. PGVV = Phaeocystis globosa virus virophage, YSLV5, 6 or 7 = Yellowstone Lake virophage 5, 6 or 7.

All the virophages are rich in K, L, E and N residues, except YsL5 is AGL-rich virus.

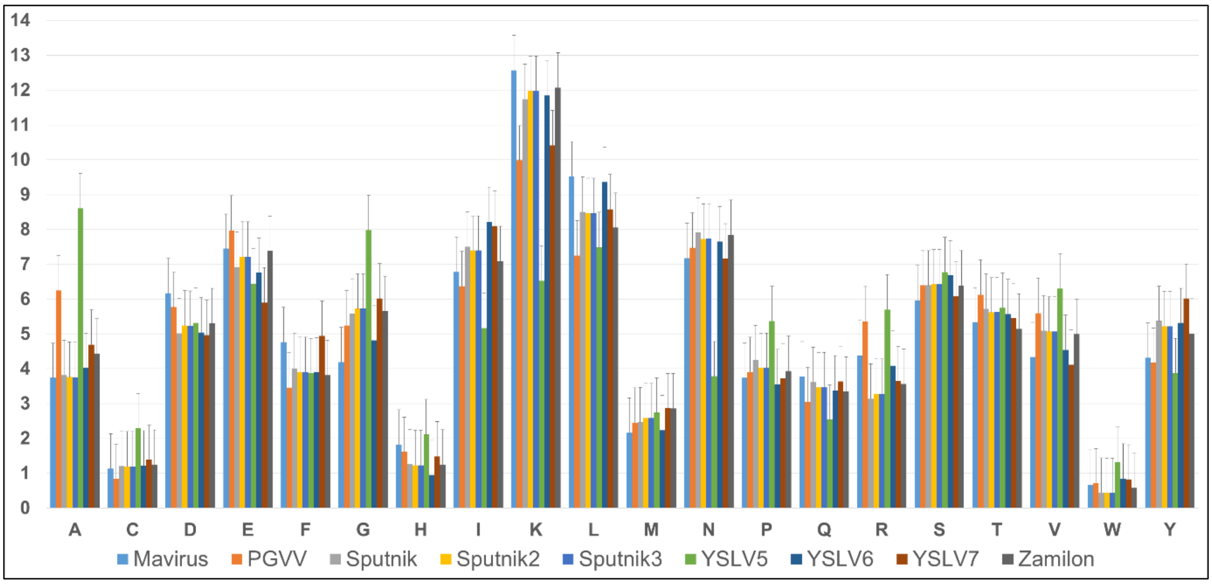

Supplementary Tables

Table S1 (Excel file). The experimentally validated functional motif table.

The table shows the enrichment of the motif-containing proteins to the proteome. The number of motif-containing proteins were counted, and then normalized to the total number of proteins within the proteome, i.e. percent to the total number of proteins. The table was used to construct Fig. 1 in the main text. For the definition of the residue symbols, see table S4.

Table S2 (Excel file). The predicted KR-rich motifs (a predicted NLS motif) in PgV using sequence search by Shetti-Motif (Sobhy 2017) and NLStradamus tools (Nguyen Ba, Pogoutse et al. 2009) (http://www.moseslab.csb.utoronto.ca/NLStradamus/).

Table S3 (Excel file). The amino acid residue coverage within each protein encoded by virophages.

The table contains (i) the coverage values of amino acid residues within each virophage protein. (ii) The average values of the residues coverages and standard deviations within the whole proteome. (iii) The final summary table of the average coverages. The largest averages are considered as the most abundant amino acids in the proteome. The table was used to construct Figure S2.

Table S4. The amino acid residue symbols used for searching functional motifs, adopted from Shetti-Motif tool, adopted from (Sobhy 2017).

**Symbol**	**Representative Motif**	**Residues**	**Physical properties**
[]	P[ED]	P, and (E or D) residues ≈ PE or PD	
{}	P{R}	P, and any residue but R	
numbers	T(2), T(2-5) or T(3,)	T is repeated 2, 2-5 or >3 times	
- “hyphen”	[ED]	E or D	Negative - Acidic
+	[HKR]	H, K or R	Positive - Basic
=	[ST]	S or T	Alcohol
*	[CM]	C or M	Sulfur containing
?	[IVL]	I, V or L	Aliphatic
&	[AGS]	A, G or S	Tiny
@	[FHWY]	F, H, W or Y	Aromatic
%	[DEHKNQRST]	D, E, H, K, N, Q, R, S or T	Polar / hydrophilic
!	[ACFGVLIPWMY]	A, C, F, G, V, L, I, P, W, M or Y	Non-polar / hydrophobic
#	[CWNQSTYKRHDE]	C, W, N, Q, S, T, Y, K, R, H, D or E	H-bond
x	Any amino acid	Any amino acid	Any amino acid
